# Errors in Figures

**DOI:** 10.1001/jamanetworkopen.2022.13901

**Published:** 2022-05-02

In the Original Investigation titled “Comparative Effectiveness of Immune Checkpoint Inhibitors vs Chemotherapy by Tumor Mutational Burden in Metastatic Castration-Resistant Prostate Cancer,”^1^ published March 31, 2022, the labels for the numbers at risk in Figure 3 were reversed and there was a typographical error in Figure 4. This article has been corrected.^1^
